# Extra-Limites

**Published:** 1840

**Authors:** 


### EXTRA-LIMITES.

### Case of Dracunculus or Guinea-Worm.

*(To the Editors of the Medico-Chirurgical Review.)*

*Kissy Hospital, Sierra Leone, 28th Nov.* 1839.

Gentlemen,—I have the honour to forward you the particulars of a case of Dracunculus or Guinea-worm which came under my care as surgeon to the Liberated African Hospital, at Kissy. Should you deem the same to possess sufficient interest to occupy a place in the columns of your valuable journal, I will feel much indebted by its insertion. I have taken the liberty at the same time of requesting your acceptance of the accompanying morbid preparation, taken from the subject of the case above alluded to.

I have the honor to be,
Your most obedient humble servant,
ROBERT CLARK,
Assistant Surgeon to the Colony of Sierra Leone.

Ogoumini, a liberated African boy, æt. 11, a native of Housu, was admitted to hospital on the 30th of January, 1839, with a worm protruding from inner and inferior third of right thigh; his general health is good. Three inches of the worm was gently drawn out, when two inches was removed with scissors and a thread attached to remainder to prevent its retreating; during its extraction he complained a good deal of pain.

Apply a soft cassada poultice so as to cover the worm.

February 4th.—Up to this date no change has taken place, but this morning the worm was found lying on poultice; it measured three feet; complains of pain in sole of left foot. On examination, I found a small vesicle, on opening which a small quantity of a milky-like fluid escaped, but no worm could be detected, the little sufferer maintains however, that there is a worm present there.

Immediately apply a large soft cassada poultice.

8th.—The poultice has been regularly applied twice a day up to this date, when a worm was observed to present where the vesicle had been opened; four inches of the animal were drawn out, when three inches were removed, the remaining portion being treated as above described, the remainder came away on 11th inst. when it was found to measure one foot and a half.

13th.—Two worms have appeared, one situated on the outer portion of left foot, the other on anterior edge of right ankle; several inches of the former were extracted and about one inch of the latter was drawn out—

℞. Liniment. Sapon. C. ℥ij.

Let the ankle be embrocated with liniment, which is also to be applied over the foot.

14th.—To day I gently drew out one and a half inch of worm situated on right ankle—that on left foot I entirely removed—says that he has a tingling sensation over the whole body, accompanied with horripulation.

20th.—The sore on left foot from which the worm was extracted has put on an unhealthy appearance, its centre being bloody.

℞. Acet. plumb. gr. vi.

Ps. opii. gr. iss.

Aquæ font. ℥j. M.

Ft. Solutio.

The lotion to be applied to sore, after which let it be covered with a poultice.

23rd.—The centre of ulcer is nearly filled up with healthy granulations.

During the night he was attacked with bilious diarrhœa.

℞. Ol. ricini. ℥ss.

Sub-mur. hyd. gr. ij.

Aquæ font. ℥i. M.

Ft. haustus, stat. sumend.

Continue lotion and dress the sore with simple cerate.

26th.—Diarrhœa has continued in spite of the administration, every three hours, of the Mist. creta. c. opio. combined with catchu, which he has taken since the 24th inst. Skin clammy—tongue red at tip—eyes are sunk in and glossy—great prostration of strength with sinking of abdominal parietes towards vertebral column, evacuations are now mixed with frothy mucus and undigested aliment—he is much emaciated. To-day a worm has appeared on the right foot, close to tarsal metatarsal articulation—it has resisted every effort made for its removal.

Continue poultices and dressing to sore on left foot, which is almost healed up. Discontinue chalk mixture.

℞. Sub-mur. hyd. ℈ij.
Ps. opii, gr. iij. M.
Ft. mass. et divide in pil. No. xij.

One of the pills to be taken every three or four hours.

His common drink to consist solely of rice water. Let the abdomen be circled with a flannel bandage, which is to be moderately tightened. To be allowed a small quantity of wine with well-boiled arrow-root.

28th.—The treatment last mentioned was regularly continued, but proved ineffectual in suppressing the evacuations, for he rapidly sunk and died on the morning of the 29th inst.

*Post-mortem Examination.*—Head—Brain healthy.

Thorax—Lung of left side adherent to the walls of chest. Pericardium contained about a drachm of serous fluid; on removing the heart and making a section through right auricle and ventricle, an organized substance of a dirty-white colour and nearly globular in form was found to fill up the auricular cavity; a band of similar colour proceeded from the tumor into the right ventricle, where it became attached to the walls of heart; the upper surface of tumor was connected to the fleshy columns of auricular portion: proceeding from inferior portion of band there was a loose process, descending into the ventricle.

Abdomen—Peritoneum contracted; stomach and intestinal canal filled with fluids of a dark green colour; the other organs were normal,

---

### AN ACCOUNT OF A REMARKABLE INSTANCE OF RUPTURE OF THE DUODENUM; AND OF SOME OTHER RARE OR INTERESTING CASES, (read at the last meeting of the British Association.) By Sir *David J. H. Dickson*, F.R.S.E. F.L.S. Physician of Plymouth Hospital.

RICHARD HAWKINS, marine, æt. 40, who had been complaining for some days, but was not considered seriously ill, at 4 a.m. on Sunday the 4th of March, whilst straining at stool, was seized with excruciating pain in the right hypochondrium. He was admitted into this hospital from H. M. S. Royal Adelaide at 3 p.m. and died before midnight. The symptoms were, severe pain in the region of the cæcum and ascending colon, which he attributed to flatulence; a hurried, restless, and impatient manner; pale, haggard, and anxious countenance, expressive of great distress; short, hurried respiration; and a very weak, quick, and irregular pulse. General depletion had been freely resorted to previously to his admission, followed by aperients, without relief; and as he attributed all his suffering to flatus, warm purgatives, assisted by turpentine enemata, carminatives, &c. were administered; followed, as the pain continued unabated, by leeching, and assiduous fomentations, but without any relief, and he sank at half-past 11 P.M. I afterwards ascertained that he had been reduced to the ranks for fighting and drinking three days previously; and while wrestling was thrown with violence backwards, on the breech of a gun.

*Sectio cadaveris,* 40 hours P.M.—The stomach and bowels were distended with flatus, and there was also some gas in the abdominal cavity. The vessels of portions of the intestines were congested; the cæcum and ascending colon were normal, but the transverse and descending portions of the gut were signally contracted. On cautiously raising the bowels, a quantity of ingesta was found to have escaped from the duodenum, which was ruptured or perforated in four different places, within an inch and a half of the jejunum. Its attenuated and pellucid appearance, especially near to its termination, rendered it probable that the mucous and muscular coats had previously undergone *ramollissement* and absorption, and that the peritoneal coat had afterwards given way from mechanical violence or distention. There was no redness nor congestion in the neighbourhood of the apertures; three of which were large enough to admit the end of the finger; and from one to two inches apart: nor did the intestines appear to have been otherwise diseased; but the internal surface of the stomach exhibited some dark red patches, from venous congestion, so well described by Dr. Yelloly in the Medical and Chirurgical Transactions. I have already alluded to the soft attenuated state of the ruptured coats of the duodenum, which was the more marked from the thick appearance of the jejunum caused by the rugæ of the valvulæ conniventes,—but there are not sufficient data from which we can fairly infer how long these lesions had preceded death; although the probability is, that they happened when the patient felt the excruciating pain while straining at stool; or whether they occurred simultaneously, or consecutively—but the situation of the openings, and the manner in which the gut is enveloped by the peritoneal reflexions, may explain why not more of its contents had escaped, in this instance; and hence persons may survive such accidents for several days, or only a few hours, according to circumstances. Looking to its very peculiar course, and the manner in which the duodenum is bound down, it does not appear to me improbable, that it should be more liable to injury from particular causes than the more free and floating intestines. I allude to violent muscular exertions, and contortions,—especially such as are accomplished by retroversion, or bending the body backwards, as in wrestling, tumbling, certain feats of horsemanship, &c. Such persons often die suddenly and without cadaveric inspection—or, should there be any examination p. m., if superficially conducted, the cause of death (as might have happened in the present case) would probably escape detection. Hence the same remark is applicable here which I made in my paper on Rupture of the Recti Abdominis and other Muscles,—read at a former meeting of the British Association,—viz. that, in all probability, death arises much oftener from such accidents than is generally imagined.

### *Case of Ileus, in which, from enormous distention, the Cæcum occupied the Situation of the Transverse Colon.*

*Richard Hoar,* S. æt. 50, was admitted from the San Josef on the 26th, with obstinate constipation of five days' standing, singultus, stercoraceous vomiting, &c. and died on the 22nd of May 1839.

*Sectio cadaveris* 21 hours P. M.—The abdomen was very much swollen and tympanitic, from its peritoneal cavity, containing a large quantity of serum, and the small intestines being much distended with flatus. Within two inches of the extremity of the ileum, a strong membranous band, probably the product of former inflammation, extended across the gut, from the lateral wall to the meso-colon, to which it was firmly attached; yet notwithstanding the lower portion of the ileum together with the cæcum, had been forced upwards into the epi-gastrium, so as to occupy the situation of, and during life to be mistaken for the transverse colon, the ileo-cæcal valve was much thickened and diseased, and nearly as hard as cartilage; and the cæcum had a black sphacelated appearance; and was enormously distended with fæces and gas, thus producing all the effects of a double strangulation. The serous and muscular tunics still retained some tenacity, but the mucous coat was quite disorganized. The stomach, liver, and also the contents of the chest were healthy, but much encroached upon by the diaphragm, pressed upwards by the unnatural position of the cæcum.

*Case of Intermittent Coma, from Diseased Brain, and Rupture of the Anterior Artery of the Cerebellum.*

*John Richards,* S. æt. 50, after having been, according to the case, in a comatose state for three days, from which every effort to rouse him had failed, exhibited symptoms of re-action, and subacute meningitis; in which condition he was admitted on the 17th and died on the 23d May, 1839. The alternations of torpor and excitement in this, as well as other cases, though the cause was organic, and progression was very remarkable: the patient, at one time lying in a low, muttering, or almost insensible state—with tremors, or alternate extension and retraction of the left limb—and apparently *in extremis*;—and at another period lying so much revived as to be able to swallow, protrude the tongue, and answer questions, though frequently incorrectly, as that he was "very well"—and to notice those around him. The similar conditions of the above artery, and of the aorta, from the deposition of osseous matter, as developed by dissection, is worthy of attention.

*Sectio cadaveris,* 18 hours P.M. The arachnoid membrane, on exposing the surface of the brain, appeared opake and milky, and was raised from its convolutions by gelatinous effusion. A considerable quantity of a bloody fluid, with semi-coagulated clots, found at the base of the brain, which further examination proved to have proceeded from the rupture of a small true aneurism of the size of a quill, and filled with colourless clots of the anterior artery of the cerebellum, about three-quarters of an inch of its origin from the basilar artery; a perforation sufficient to admit the probe having taken place in its anterior aspect. The coats of the artery presented distinct ossific deposits, feeling gritty internally; the left side of the cerebellum was wasted, soft and diffluent, and resembled the appearance of curdy pus; particularly over the seat of the arterial disease, which may probably be attributed to the pressure of the effused blood. The lateral ventricles were distended with bloody serum; but the brain was in this case natural. The lungs were healthy—the heart slightly dilated. The aorta was sacculated, and its elasticity impaired by extensive ossific deposition, presenting bony scales, as large as a sixpence, and easily detached. The tricuspid valves were thickened, but the rest of the vascular system, as far as examined, appeared to be normal. The abdomen did not present any change worthy of notice.

*Joseph Rowe,* was another case of *arachnitis,* during last quarter, which besides congestion, subarachnoid effusion, serum in the ventricles, &c. exhibited a very extensive deposit of little semi-cartilaginous bodies, in the sub-serous cellular tissues of the diaphragm, stomach, spleen, mesocolon, &c.; they formed small, generally round, well-defined spots, or patches, and were confluent like small-pox all over the surface of these organs. While on this subject, I may briefly notice that in a patient named *Malcolm Marshall,* recently under surgical treatment, for an abscess of the thigh, nearly well—and who had not betrayed any previous indications of cerebral disease, comatose symptoms unexpectedly appeared, and terminated fatally in a few days. Upon dissection a portion of the upper and anterior part of the posterior lobe of the right hemisphere, appeared like a red, vascular mass from the effusion, or exhalation of blood from numerous small vessels; and to the anterior extremity of the right olfactory nerve, which was much thicker than natural, a tubercle of the size of a large pea, and maturating in the centre was attached; while a similar body, but still larger, was imbedded in the optic thalamus of the same side. Tubercles were also interspersed through the lungs, which adhered to the costal parietes; and from similar depositions in the sub-serous tissue, the intestines appeared studded, as if with a thick crop of small pustules; which proved to be numerous tubercles of a yellowish colour, elevating the peritoneal coat. The upper surface of the liver, which was intimately adherent to the diaphragm, also exhibited numerous patches of tuberculous depositions.

*Case of Phthisis with the Foramen Ovale open.*

This was one of those instances in which the communication between the right and left cavities of the heart had continued unclosed, without producing *cyanosis;* and in which state, indeed, the pensioner had completed his allotted period of servitude in the marines, and had attained the rank of sergeant; and, so far as I could learn, without having suffered any particular inconvenience; until from the increasing size, and weakness of the right auricle, with the adherent, and very condensed state of the diseased lungs, the functions of circulation and respiration became more and more embarrassed. The symptoms were, cough, increasing dyspnœa, with a puffing or blowing motion of the lips (which were not discoloured) during respiration; anorexy; emaciation; great prostration and faintness; latterly approaching to syncope, on attempting to get up; with the pulse generally about 100, but very weak, irregular, and intermitting. These distressing feelings were much relieved by wine and stimulants; but especially by the sustaining power of (the muriate of) morphia; and he always expressed himself much revived and comfortable after having taken the anodyne draught.

*Sectio cadaveris,* 20 hours P.M.—The lungs, on both sides, were universally adherent, and studded with crude tubercles, particularly the right lung, which was besides so much congested with bloody serum, and so condensed in structure, as to weigh 2lb. 11oz., while the left weighed 1lb. 8oz. The heart was rather larger than natural; the walls of the right auricle, which was enlarged, were remarkably thin, and the septum which ought to have closed the *foramen ovale* was found to be partially open and cribriform in its anterior part. One of the openings was sufficiently large to admit the top of the little finger. The valvular apparatus did not seem to be abnormal. The abdomen contained some turbid primrose-colored fluid, and the intestines were agglutinated together by lymph, which was effused in large quantities in the pelvis. Incipient ulceration existed in the ilium. The stomach was large, as was likewise the liver, which was pale and friable, and weighed 4lb. 9oz. The gall-bladder was much distended, the kidneys were pale, the body was emaciated, and the limbs œdematous.

*Case of Phlegmonous Erysipelas remarkable for the great rapidity and extent of Disease in a short period.*

John Duncan, S. æt. 38, who was stated to be then in his usual health, had pulled from the flag ship, in harbour, to Plymouth Sound, in one of the cutters, on Thursday, the 4th April—went on shore, on leave, afterwards—was seized the same evening with severe pain in the left arm, which soon extended to that side of the chest—was prescribed for by the surgeon on Friday, and brought to the hospital on Sunday, at 11 A.M., in a dying state, and expired early next morning. The symptoms on admission were, great dyspnœa and pain, with increasing tenderness and tumefaction of the left arm and side of the chest, cough, mucous rattle, cold clammy skin, pale countenance, great anxiety and restlessness, and a very weak pulse; which sunk so rapidly as soon to be imperceptible at the wrist. Any measures which could then be attempted were of course only palliative, and afforded but little relief; and, after a night of great suffering and restlessness, he died on Monday, at 5 A.M.

*Sectio cadaveris*, 30 hours P.M.—There was much diffused swelling above the clavicle, and extending downwards, as far as the lower margin of the great pectoral muscle, yielding a doughy feeling of indistinct fluctuation, as if from some fluid effused in the subcutaneous and intermuscular cellular tissues. On the first incision, purulent infiltration of the tumid parts became evident; and its extent was then carefully traced by Mr. Male. The loose reticular membrane connecting the cervical and brachial plexuses of nerves was saturated with pus. The disease pursued the course of the carotid artery and jugular vein, as far as the thyroid cartilage; extended under the clavicle; surrounded the subclavian vein; burrowed between and under the pectoral, subscapular, and latissimus dorsi muscles; and followed the course of the brachial vessels and nerves nearly as far as the elbow-joint. There was œdema, but little or no external discoloration during life, to indicate such extensive destruction of parts. The pectoral muscles were paler, and more easily lacerated than the unengaged ones. The thorax was then opened—the left lung appeared collapsed, and the secretion of some gaseous, as well as nearly a pint of bloody sero-purulent fluid had taken place in the cavity of the chest. The pulmonary costal and diaphragmatic pleuræ were intensely inflamed; but the lung which adhered slightly posteriorly was not much congested. The liver was large and engorged with blood.

*Case of Chronic Inflammation and Scirrhus of the Pylorus and Pancreas—with Ulceration of the Duodenum, &c.*

Mr. James Keating, æt. 25, who had returned from the West Indies on the 30th of June, since which time he had been under the care of a private practitioner, was admitted into this hospital in the last stage of atrophy, from constant vomiting, of a dark bilious-looking fluid, on the 29th July, and died on the 1st August. Neither medicine nor nourishment could be retained; and it is needless to say, not the slightest hope of his recovery was entertained upon his admission. He had been subject to vomiting two or three times a day, and confined to his cot from May until the middle of October 1838, during which period he was reduced to the utmost degree of debility and emaciation, when he was sent to the hospital at Havannah. Soon after he rejoined his ship. On the 7th of November the vomiting returned; and during the passage homewards recurred generally twice a day; and since his arrival it had continued without intermission.

*Sectio cadaveris*, 18 hours P.M.—The whole body was reduced to a state of extreme atrophy. The contents of the thorax were perfectly healthy. On opening the abdomen, the large size and streaky appearance of the stomach, and the turgid state of the intestinal veins attracted attention. The stomach, which contained a quantity of fetid dark green fluid, was hypertrophied, and extensively ecchymosed, especially towards the pylorus, which was thickened, but not contracted. The whole of the mucous surface of the duodenum, as far as the opening of the biliary and pancreatic ducts, was the seat of destructive ulceration and greenish sloughs; and immediately below this part, it suddenly contracted so much as scarcely to admit the little finger; but below, it reassumed its natural appearance. It was removed with great difficulty in consequence of its firm connexion with the pancreas, which was so indurated as scarcely to allow the knife to pass through its structure; while its greater extremity was so much enlarged as to displace the duodenum forward. The pancreatic duct, which was nearly as large as a goose-quill, and the radicles, were filled with pale thick pus; and several cells contained calcareous deposits in shape and size as large as peas. This mass of disease compressing the vena portæ accounted for the turgid state of the intestinal veins. The liver appeared healthy; but from the difficulty of emptying itself, the gall-bladder was distended to the size of a large winter-pear —its contents were thick, and of a hemlock green colour, not like healthy bile. The cystic was distended as far as its entrance into the common duct, where some obstruction had taken place. The spleen, kidneys, &c. were natural, and the mesenteric glands were not much enlarged.

*Case of Peritonitis and Scirrhoma.*

Although disposed to believe the heterologous formation to be described in this very curious case, (and which I have called Icthoid, from its resemblance to fish in colour and general appearance,) to be of constitutional origin, and its *locale* to be chiefly in the subserous filamentous tissue—and therefore in deference to the eminent authorities of Dr. Carswell, and other pathologists, I have classed it as a variety of *scirrhoma,* yet I do not feel altogether satisfied of the pathological correctness of doing so, or, at least, if rightly so classed, of grouping as mere varieties of the specific divisions many anomalous growths, differing in anatomical and physiological characters, and in malignancy, under the generic term of *carcinoma:* but as I have neither space to adduce my reasons, nor have had sufficient opportunities of investigation, to entitle my doubts upon the subject to much attention, I shall proceed, without further remark, to detail the remarkable case in question.

*Robert Burden,* marine, æt. 32, was admitted from Pembroke Yard, on the 27th October, and died on the 26th December, 1837. He had suffered from influenza in the Spring, followed by severe dyspepsia, gastro-enteric pain, and progressive emaciation. The chief symptoms were great abdominal tenderness, and, except when under the influence of opium, that unhappy yearning, discontented look, so indicative of visceral disease; the pulse weak, and generally about 100; complete anorexy, and frequent sickness, but apparent inability to vomit; torpid bowels, with such extreme atrophy as to resemble, if not surpass, in extenuation "L'Anatomie Vivante;" indeed, latterly, when he could not lie down, with the eyelids closed, and a spot of hectic on the cheek, he might be compared to a sitting painted corpse.

*Sectio cadaveris,* 32 hours P.M.—Besides the body being so atrophied, the eyes were sunk in their orbits, and sloughing of the cornea, &c. had commenced. A large rose-formed warty excrescence protruded from the navel, but did not appear to have any vascular connexion with the diseased parts within. There was a large quantity of fluid in both cavities of the thorax and abdomen, especially the former; but with the exception of slight pleural adhesion, the lungs, pericardium and heart, appeared to be healthy. In the abdomen the ravages of disease were manifested to an extraordinary extent. The parietal and visceral reflexions of the peritoneum exhibited a dark red, or purple, ecchymosed appearance; mottled with small white spots; and the intestines were agglutinated by the effusion of lymph. Indeed the stomach, liver, pancreas, colon, &c. were accreted into one mass, defying intelligible description. The stomach was contracted into deep rugæ, and marbled by tints of a yellow, vivid, and deep red, and black colours, and its capacity greatly diminished by the density of its coats, caused by a copious deposit of a scirrhous or semicartilaginous appearance. It was most abundant at the great end, and in the large intestines; the walls of which, and the duplicatures of the peritoneum, were much thickened by this morbid deposition. In a less, or earlier stage, it also existed in the small, but to a much greater extent in the large intestines. The liver was congested, and the spleen small, but the internal structure of these organs and of the kidneys appeared normal. On making a transverse section of the large intestines, and especially of the descending colon, the calibre of the gut appeared much contracted, as already observed; being encircled by a broad ring of the same dull-white, yet glistening fish-like substance, resembling skate, but fibriform, and of a firmer consistence, and which varied from half an inch to upwards of an inch in thickness. Except slight intervening traces of tuberculous matter between the mucous and muscular coats, they seemed to be healthy, and their cohesive and cellular connexion unaffected; but these subjacent coats were so completely disconnected from the serous coat, by the interposition of this icthoid product, that large portions (I might even say yards,) of the former could be drawn out from the latter with the utmost facility: and, taken altogether, this was perhaps one of the most extraordinary cases on record.

Royal Hospital, DAVID J. H. DICKSON.

Plymouth, 22d August, 1839.

---

### Carbonate of Iron as an Antidote to Arsenic.

*(To the Editors of the Medico-Chirurgical Review.)*

Gentlemen,—Being a subscriber to your valuable periodical, since 1820, I have had an opportunity of becoming acquainted with the principal discoveries connected with medico-chirurgical science, as well as the opinions of the leading members of it, for which I take this opportunity of offering you my small tribute of acknowledgement.

I was aware of Dr. Bunsen of Gottingen's plan of administering the hydrated peroxide of iron as an antidote to arsenic. I have also read the case of M. Deville, successfully treated by the tritoxide of iron and the experimental researches on the oxides of iron from the Revue Medicale.

As the two following cases, which have fallen under my own observation and management, may prove useful in farther illustrating the effects of iron, as an antidote to arsenic, I think it may be of some practical benefit to have them generally known, and if you think them worth publishing you are at liberty to do so.

*Case* 1.—J. Davis, aged 12 years, brought by his mother to my surgery, April 26, 1839, twenty minutes after eight A.M. having swallowed, by mistake, about a teacupful of a solution of white oxide of arsenic, which had been prepared to cure the itch, with which some of the family were afflicted.

*Symptoms.*—Violent retching and vomiting, (he fortunately had taken his breakfast just before.) Excessive thirst, and intense sensation of burning in the throat and stomach; breathing difficult; great tremor of the whole body and inability of controlling the muscular action of the limbs.

*Treatment.*—℞. Ferri carbon ʒij. p. g. acaciæ ʒj. aquæ Oss.* ft. haust. s.s. Immediately rejected. Ferri carb. cretæ pptæ āā ʒij. p. g. acaciæ ʒj. aq. et liq.

---

* The reason of administering so large a quantity of water, was to diffuse the antidote over a larger surface of the coats of the stomach.

calcis āā ℥iij. were retained in the stomach. He took four such doses, but after the third, all symptoms of poisoning ceased. The lad drank the solution out of the bottle, which was previously full, and contained ℥j. of oxide of arsenic and about ℥xxiv. of water. Water, I believe, at the common temperature, will dissolve about $\frac{1}{100}$ of arsenic, and I suspect the boy might have swallowed about a scruple of the oxide in the teacupful he took.

*Case* 2.—Mr. T. O. (a respectable tradesman,) *thinking* he had the itch, (which he had not, but a common eruption to which he is subject every Spring,) washed his body and limbs very freely, on the evening of May 8, 1840, with a strong solution of arsenious acid and sulphate of copper, but he did not know what the solution was, as it was given to him by a *friend.* Saw him about eleven o'clock A.M. of the 9th, complaining of thirst, dryness of the fauces, great uneasiness of the stomach and abdomen, and at times of a burning sensation and acute pain in the stomach; pulse hard and about 70; bowels constipated, and general uneasiness and restlessness.

*Treatment.*—V. sectio ad ℥xviij. and prescribed magnes. sulph ℥iss. d. aloes co. et ess. sennæ āā ℥ss. aquæ ℥viij. Two table spoonsful of which were to be taken every four hours with three of warm water, till free action of the bowels was produced. At about five o'clock, P.M. found him with all the symptoms much more intense, with the addition of retchings; great soreness in the stomach; anxious countenance; in short, with all the symptoms of poisoning by arsenic. Directed to drink decoction of flax-seed very freely, and to take immediately, ferri carb. ʒi, p. g. acaciæ ʒss. in thick gruel, with directions to repeat the dose in half an hour, and then every hour till quite relieved. About two minutes after the first dose, his throat and mouth became moist; the pain ceased entirely; he dropt asleep, and slept soundly all night. Of course took no more medicine, and was quite well the following morning.

The subjects of these two cases are now living in health, and are evidences of the action of carbonate of iron in cases of poisoning by arsenic. In country towns and villages, the hydrated peroxide of iron cannot be obtained. Even in this, which is a considerable market town, we have no practical chemist, and the nearest is Messrs. Blunt, of Shrewsbury, 18 miles distant. Carbonate of iron is kept by every practitioner and druggist, and if future trials prove as successful as the above two described, the antidote will be within the reach of every one.

I have the honor of remaining,
Gentlemen,
Your very obedient and very humble servant,
P. L. SERPH,
Surgeon.

Welshpool, 15th May, 1840.

---

### Soupe a la Minute.

*At page* 239 *of No.* 59, *January,* 1839.

Six pounds of butter, &c. &c. &c. fried with a handfull of onions and the same quantity of *aulx,* you inquire what are these? *Aulx* is the plural of the word *ail* (garlic).

### RECLAMATION.

### DR. MURPHY TO THE EDITORS OF THE MEDICO-CHIRURGICAL REVIEW.

WE give insertion to the following Reclamation, because we see in it none of those offensive personalities, those effusions of spleen, nor those petty out-breaks of wounded vanity too common in the present days of periodical literature. Dr. Murphy argues his case, and though we cannot coincide with him, and could, we conceive, show satisfactory reasons *for* our own opinions and *against* his, yet we give him the benefit of the last words. As he had the first, we think he has every reason to be satisfied; and if truth be on his side, with such logical advantages, he *must* be triumphant. Dr. Murphy, and indeed every author, may feel assured that in us he will meet with a liberal, we should not be going too far did we add—a generous critic. We feel nothing but disgust at the carping jealous spirit too frequently evinced by journalists.

In the Medico-chirurgical Review of October, 1839, a small work of mine, on "Mercury being the Cause of Secondary Symptoms," was reviewed. The doctrines then advanced being novel, and opposed to those generally received, a severe investigation of their correctness was expected and desired. The high character which your Journal has gained both for medical knowledge and impartiality was then evidenced as usual, and I regretted that the criticism was unfavorable. Had your censure been less severe, many practitioners might have been induced to put them to the test of experiment, and the result, I am still convinced, would have confirmed my views. A very eminent practitioner in this town told me that his experience had led him to adopt a treatment nearly similar to mine. In the seventh volume of the Transactions of the Provincial Medical and Surgical Association, page 238, Dr. Otto, of Copenhagen, states, "The results which were published proved the value of the method even beyond expectation, so that I now never use mercury against syphilis, effecting a complete cure of all its forms within a much shorter time than was formerly done by mercury, and *without seeing secondary symptoms arise afterwards.* Professor Windt, Dr. Muller, and Dr. Svitzer, have been equally successful with this mode of treatment, though they think that there are forms of syphilis which require mercury, and consequently now and then employ it. *I frankly confess that I have not seen such cases.*"

There is here the testimony of a most eminent Danish physician, who is simply relating the result of his observations, and many years cannot elapse without the truth of the doctrine being universally admitted. The review, I suppose, was written by the junior Editor, who, I understand, is peculiarly qualified for the task, and his affording nine or ten pages for its examination is highly commendable, especially when it is remembered that other journals would scarcely examine the doctrine. The first objection is, that there is no positive proof adduced in favor of the identity of the gonorrhœal and syphilitic poisons in consequence of the vague definition of chancre. Now I have defined chancre according to Hunter, and I really know not what stronger proof can be urged, or one with which I myself would be more fully satisfied, than if an irreproachable female newly-married is found with discharge from the vagina, painful micturition, genuine chancres and buboes, from which symptoms she was previously free, while her husband, who is anxious for her recovery, and will not on her account deceive his physician, tells him that he had gonorrhœa just previous to marriage, which again shewed itself immediately afterwards, and that on a close inspection of himself no trace of a breach of surface ever having existed can be found, I have seen several cases nearly similar, and I wish to ask any medical practitioner to what other conclusion could I have come, or what interpretation he can put on such occurrences.

The reviewer also asks what analogy can there be between specific chancre and excoriation of the cheek? The comparison was made for the purpose of proving, beyond any cavil, that a non-specific secretion may cause an ulcer, and that this healthy ulcer may produce suppuration of a gland, and that in a similar manner gonorrhœa may produce an ulcer, and this ulcer may be attended with buboes, without there being any thing specific either in the gonorrhœa, the ulcer, or the bubo.

Experiments, however, as you justly remark, are called for, but few will be found to submit to such experiments; and they would be unjustifiable without the consent of the party, and without their nature being explained.

On chancre I have very little to remark. The facts I chiefly wished to lay before the profession have been assented to. The specific nature of a sore with callous edges and base, so strongly maintained by Hunter, and so implicitly confided in by many practitioners, is ridiculed.

That any other ulcer may present the same appearance is allowed, but that the structure of the part modifies the appearance of an ulcer is almost denied. The impression on my mind still is, that the structure changes the appearance of a sore, but perhaps I may be mistaken. Ulcers on the pubes, although their bases and edges are hard, have their edges raised, which is not the case with ulcers on the glans penis. That the structure gives a peculiar character to an ulcer, and requires a peculiar mode of treatment, is borne out by a very recent case. A healthy man, aged 30 years, had an ulcer, of 12 months' duration, in the right angle of the lips; it was excavated, foul, and irregular, it had eaten away to the size of a sixpence on the inside of the upper part of the cheek, and nearly to the same extent on the lower, the peculiar hardness of the edges of the ulcer, which were partly formed by the labiæ, struck me as being similar to chancre, a form of ulcer which soon yields to mercury. A gentle salivation was excited, and the sore immediately healed.

That bubo may and usually does arise from irritation, is also conceded. To prove that it never arises from absorption, I will not attempt, as I do not mean to deny it. But if it be absorption, and not irritation, that causes a venereal bubo, it is difficult to account for the following facts. The venereal poison enters the circulation—it must, therefore, be present in every gland of the body, and must have passed through the second order of glands. Why, then, do no glands enlarge except those of the first order?

There has been no answer given as to how matter taken from a venereal bubo causes no disease in a healthy subject, while it produces, or rather is said to produce, such a train of symptoms in the affected individual.

It must also be remembered, that the variolous and vaccine matter enter the circulation without producing any enlargement of a lymphatic gland. In scarlatina no glands enlarge except those of the neck, and on inspection there will be found ulceration of the fauces.

Ulceration of the throat is also admitted to be an effect of mercury, but it is insisted that it is also produced by venereal, and because the ulcer will sometimes be very obstinate until mercury is used. This is regarded as an irresistible argument in favour of its syphilitic origin; but mercurial rheumatism sometimes will yield to no other remedy. My argument is, that mercury leaves the constitution peculiarly liable to be acted upon by cold, the throat inflames, and the consequent ulceration cannot be healed until the inflammation be subdued. This we daily witness in sore legs. Mercury relieves the inflammation, and cicatrization commences.

I have no recollection of ever having met chronic ulceration of the throat which was not healed by other remedies. If a large ulcer perforates the velum palati, without extending to its free margin, it will almost immediately commence healing on the division of the bridle: but unless the ulcer be very large, there is an objection to this mode of treatment, as the voice usually becomes nasal.

There is however one important admission on the part of the reviewer which must tend to a more exact knowledge of this disease, if acted upon by the profession. It is, " That he has no hesitation in expressing his opinion, that nodes are in the immense majority of instances the result of the abuse of mercury." He does not advocate the whole of my doctrine on this point, but refers to one case of syphilitic origin in a gentleman from Edinburgh, who could not, unless a professional gentleman, be positive whether mercury had been used or not, and who ought to have been asked whether he had taken mercury for any other complaint. He has argued, in the same page, that an ulceration of the throat which cannot be healed until mercury be exhibited, must have a syphilitic origin, and yet he himself immediately afterwards declares, ex cathedrâ, that nodes are mostly the result of mercury, whereas there is no practitioner but must acknowledge, that of all diseases, there is not one which more rapidly yields to this remedy, or which more obstinately resists any other. But these contradictions must continually occur even in the best informed practitioners, until the plain simple view of the disease be taken which I have pointed out, and which is now acted upon by the Danish physicians.

That mercury should be altogether proscribed in the treatment of syphilitic complaints is a doctrine which I have never advocated, on the contrary, I stated that both chancres and buboes are more speedily removed by it, than by any other remedy. I only wished to caution practitioners against confounding the effects of a remedy with the symptoms of a disease. During the last year I have treated several cases of syphilis without mercury, but I have not met with a secondary symptom, while those under the care of other practitioners, where mercury has been used, have occasionally come under my observation.

If the following fact is not explicable on the doctrine of mercury being a cause of secondary symptoms, I know not how it can be otherwise interpreted.

June, 1840, Henry Ashold, of the Old Swan, West Derby, presented himself at the Norton-street Institution, for diseases of the skin, with
and iritis of both eyes. The appearances were the same as those termed secondary symptoms. The patient was seen by Messrs. Ashcroft and Martin. His history of the case was, that two months previously he was under the care of Mr. Swinburn for rheumatism, which was cured by salivation. The eruption appeared after having been exposed to a shower of rain. He never had syphilis. When I meet with such cases, and on the other hand, find that the non-mercurial treatment of syphilis is not followed by any peculiar symptoms, I feel that I am entitled to have the doctrine investigated more closely. It is true, I am met by the reviewer, who states that *he has seen* sores give rise to secondary symptoms. The fact of course I will not contradict, but if the man was treated in a Lock Hospital on the non-mercurial plan while others in the same ward were under the mercurial treatment, his system was exposed as much to the chance of being mercurialized as those persons who become salivated by using mercury in some of the arts, and therefore such cases must not be brought forward ; or if a person be treated for a chancre which heals under a mercurial plan, and in a few months suffer from a similar disease which is cured by the non-mercurial treatment, and in some months afterwards, secondary symptoms appear, they ought not be laid to the account of the latter treatment. Cases of the latter kind must have been very common amongst soldiers, on whom Mr. Rose made his first observations.

Cases of the following kind are by no means rare. A person subject to ulceration of the throat, is liable to its appearance on exposure to cold, if he contract a chancre, and no mercury be used, the ulceration on its next return is set down as a genuine secondary symptom. Many practitioners still act on theerroneous aphorism of Boerhaave, " In dubiis suspice luem."

### Solutio Magnesiæ Bicarbonatis.

*(Mr. Dinneford's Reclamation.)*

The Solubility of Carbonate of Magnesia in Distilled Water, impregnated with an excess of Carbonic Acid, has been long known to both Scientific and Practical Chemists. We might refer to Authors at hazard for this, but the following examples will be sufficient to establish the fact. In Fourcroy's Chemistry, published in 1790, vol, 1, page 273, the author states, it is "dissolved in water, saturated with aërial acid."—In the System of Chemistry, published at Edinburgh, in 1809, by the late celebrated Dr. Murray, the following sentence will be found at page 533, vol. 2: "When acted upon by water impregnated by Carbonic Acid, it (Magnesia) is dissolved."—In Thompson's Inorganic Chemistry, published in 1831, vol. 2, page 532, it is stated, "Carbonate of Magnesia dissolves in water impregnated with Carbonic Acid."—In Brande's Manual of Chemistry, published in 1836, page 627: "When a current of Carbonic Acid Gas is passed through a mixture of water and Magnesia, a clear Solution of Bicarbonate of Magnesia is obtained." The above works are the text books in the hands of every Student of Chemistry. They are written by the most celebrated Chemical Philosophers and eminent Teachers of the last half-century. Is it not surprising then, that at the present day, any person should pretend to have discovered so well known a fact? I make no claim to such a discovery, but I have used it, and shall continue to do so.

I am well aware how little importance is attached by the public to disputes, engendered and fostered by the clashing pecuniary interests of rival manufacturers and tradesmen, but a few words are due to my friends in answer to the accusations circulated in anonymous handbills (without even the the printer's name attached to them), as well as anonymous letters.

In the course of the year 1838, I was engaged in a series of *laborious and costly* experiments for discovering the most effectual means of obtaining a PURE and PERFECT SOLUTION OF MAGNESIA; and in the month of November I offered my Solution for sale to the public. Sir Jas. Murray, who had been employed on the same subject, called upon me on the 23d of December, and proposed terms of agreement between us, by which he was to surrender to me the sale of this preparation in England, whilst he reserved to himself the market of Ireland and Scotland; knowing but little of Sir James Murray *at that time,* I was ill-advised enough to listen to his proposals. Instructions were then given by Sir J. M. to an attorney to draw up articles of agreement between us.

What was my astonishment to find, that at the very moment when the lawyers were receiving those directions, the newspapers were filled with advertisements for the sale of the fluid magnesia in London. At first some apprehension seems to have been entertained by the author of these advertisements, for they were issued in the name of the "Successor of the Inventor," "Discoverer of the Process" and other disguises. By degrees, however, growing bolder, the name of "E. Murray, Chemist, 44, Regent Circus," (where no Chemist resides, the house being occupied as a Steam-Packet Office); "of Mr. B. Murray;" then of "Dr. Murray, *chemist,* 33, Piccadilly," which happened to be the residence of a respectable stationer); and at last, when all these subterfuges were exposed, the name of Sir James Murray was inserted in the advertisement. In this way, no doubt, it was the intention to amuse me by affected negociations with lawyers, while the most active measures were taken for a premature occupation of the market.

Soon after my Solution of Magnesia became known to the medical world, I received an unsolicited testimonial of its value from that eminent physician, Dr. Conquest. Though I have received many such since, this at the time was very gratifying to me, and I made it public. I had no sooner done so, than Sir Jas.

Murray, in great alarm, called upon Dr. Conquest, and made such an impression upon his mind, that he expressed regret at having written my testimonial. But Dr. Conquest, in consequence of a *very natural suspicion* that an unfair statement had been put before him, cautiously guarded his remarks with the following significant sentence :—" I SAY THIS ON THE ASSUMPTION THAT ALL YOU HAVE SAID TO ME IS BASED UPON TRUTH."

It will not surprise any one, after the foregoing statement, to learn that Sir James Murray has published that letter without the ***qualifying sentence,*** which removes its sting.

Dr. Conquest has since expressed his regret that he should have allowed himself to be imposed upon by an *exparte* statement of Sir James Murray, and says, " I HAVE ENQUIRED INTO THE CIRCUMSTANCE, YOU ARE AT LIBERTY TO CONTINUE THE USE OF THE CERTIFICATE I GAVE YOU."

The following is a copy of it :—

" Dear Sir,—I have been much pleased with the Bicarbonated Solution of Magnesia, and feel with many others, that the profession and the public are indebted to you for a highly valuable addition to our list of medicines. As an agreeable mild aperient it cannot fail to supersede many now in use, but which so offend the taste and the stomach, as to justify their banishment from our prescriptions. " Yours, respectfully,

" J. T. CONQUEST.

" Mr. Dinneford, 172, Bond-street. " Finsbury-square, July 18, 1839."

A pamphlet has lately been published by Sir James Murray, in which he attempts to support his pretensions by heaping calumnies, which he purports to be written by different persons. Among them is a Mr. Murray, whom he styles Lecturer on Chemistry at Hull. That gentleman has written to me, and after denying in the strongest terms the impudent fabrication attributed to him, says, " In proof of my sincerity, I have written to Sir James Murray to cancel my name *toto cœlo* in connexion with his testimonials."

Mr. Clarke, Surgeon, of the Hampstead Road, with whose name a similar gross liberty has been taken, has written an indignant denial of the offensive calumny attributed to him by the same person.

Mr. Herron, of the National Medical Hall, Dublin, has written to me, and says, " You will see by the enclosed, Sir James Murray has removed *my name* from the certificates given me, and substituted *his own ;* he has broken his faith with me in every way, I shall therefore be happy to undertake your agency."

After this exposure of his recklessness of assertion, the public will know how to estimate the various productions emanating from this person. All future attacks and calumnies I shall pass without comment. My friends will easily believe that the man who can act in the manner I have related, will invent stories to justify the language he has employed. I cannot say more of them than I have have already said—that all his observations are scandalously false. That which is of real value to the public is the admission of Sir James Murray himself, that *" the process had been greatly improved since his time by persons in London."** These improvements are my discovery; they have cost me great pains and much thought. I have not only *improved* the process, but I have *exhibited it* to many of the most distinguished members of the Medical Profession in this country, who have gratified me with their thanks, as my numerous certificates will show. I offer my Solution of Magnesia to the Public in the utmost state of PURITY, ***and I know it is,*** and WILL BE APPRECIATED.

CHARLES DINNEFORD,
Family Chemist to her Majesty the Queen Dowager,
and His Royal Highness the Duke of Cambridge.

173, *New Bond Street.*

* Copied from the *Dublin Medical Press,* Jan. 9, 1839.

N. B.—Mr. Dinneford begs to announce, that in consequence of the great improvements he has effected in his machinery, and the employment of a powerful steam engine for condensing the Solution of Magnesia, he is enabled to supply that preparation for the purposes of dispensing at a reduced price. It will therefore, in future, be sold in stone bottles containing 5lbs. at 5s. 6d. each, including the bottle. This low price will prevent the necessity of increasing the ordinary charges for medicines.

---

